# The Influence of Soil Fertilization on the Distribution and Diversity of Phosphorus Cycling Genes and Microbes Community of Maize Rhizosphere Using Shotgun Metagenomics

**DOI:** 10.3390/genes12071022

**Published:** 2021-06-30

**Authors:** Matthew Chekwube Enebe, Olubukola Oluranti Babalola

**Affiliations:** Food Security and Safety Niche Area, Faculty of Natural and Agricultural Sciences, North-West University, Private Bag X2046, Mmabatho 2735, South Africa

**Keywords:** phosphorus cycle, soil fertilization, maize rhizosphere, shotgun metagenomics

## Abstract

Biogeochemical cycling of phosphorus in the agro-ecosystem is mediated by soil microbes. These microbes regulate the availability of phosphorus in the soil. Little is known about the response of functional traits of phosphorus cycling microbes in soil fertilized with compost manure (derived from domestic waste and plant materials) or inorganic nitrogen fertilizers at high and low doses. We used a metagenomics investigation study to understand the changes in the abundance and distribution of microbial phosphorus cycling genes in agricultural farmlands receiving inorganic fertilizers (120 kg N/ha, 60 kg N/ha) or compost manure (8 tons/ha, 4 tons/ha), and in comparison with the control. Soil fertilization with high level of compost (Cp8) or low level of inorganic nitrogen (N1) fertilizer have nearly similar effects on the rhizosphere of maize plants in promoting the abundance of genes involved in phosphorus cycle. Genes such as *ppk* involved in polyphosphate formation and *pstSABC* (for phosphate transportation) are highly enriched in these treatments. These genes facilitate phosphorus immobilization. At a high dose of inorganic fertilizer application or low compost manure treatment, the phosphorus cycling genes were repressed and the abundance decreased. The bacterial families *Bacillaceae* and *Carnobacteriaceae* were very abundant in the high inorganic fertilizer (N2) treated soil, while *Pseudonocardiaceae, Clostridiaceae*, *Cytophagaceae, Micromonosporaceae*, *Thermomonosporaceae*, *Nocardiopsaceae, Sphaerobacteraceae*, *Thermoactinomycetaceae, Planococcaceae, Intrasporangiaceae, Opitutaceae*, *Acidimicrobiaceae*, *Frankiaceae* were most abundant in Cp8. *Pyrenophora*, *Talaromyces*, and *Trichophyton* fungi were observed to be dominant in Cp8 and *Methanosarcina*, *Methanobrevibacter*, *Methanoculleus*, and *Methanosphaera* archaea have the highest percentage occurrence in Cp8. Moreover, N2 treatment, *Cenarchaeum*, *Candidatus Nitrososphaera,* and *Nitrosopumilus* were most abundant among fertilized soils. Our findings have brought to light the basis for the manipulation of rhizosphere microbial communities and their genes to improve availability of phosphorus as well as phosphorus cycle regulation in agro-ecosystems.

## 1. Introduction

Driven by the need to increase crop yield, farmers worldwide have resorted to employing either organic or inorganic fertilizers to boost soil fertility. Among other nutrients, phosphorus is an essential nutrient for plants and soil dwelling microbes. Phosphate bearing rocks are used in the production of inorganic fertilizers, but this source of phosphorus is gradually depleting, making its availability over a long period of time a major concern. Although fertilizers containing phosphate are being added to the soil, its loss through runoff generally has serious consequences to water quality [1,2,3].

Nevertheless, the reactivity of phosphate ions through redox reaction in the soil makes its availability very limited, and only a minute quantity is accessible to plants for absorption. The rest is either immobilized in the soil or leached away [4,5,6] causing phosphorus starvation to both soil dwelling microbes and plants. Bioavailability of phosphorus in the soil are influenced by both biotic and abiotic factors. At the biotic level, microbes capable of producing organic acid through carbon metabolism as well as the synthesis of hydrolytic enzyme such as phosphatase are capable of phosphorus mineralization and solubilization [7,8]. Plants, on the other hand, influence phosphorus availability by manipulating the chemistry of the rhizosphere through root exudation, mycorrhizae formation, phosphatase and organic acid production [9,10,11,12]. Moreover, abiotic factors like drying and rewetting of soil, temperature, pH, and soil depth [13,14] determine the extent of phosphorus availability in the soil and its reactivity. Be that as it may, a study by Luo et al. [15] has shown that biotic factors affect phosphorus availability in the soil more than the abiotic conditions. The biotic components of the soil possess phosphorus metabolizing genes, which enhance their extraction of immobilized, organic, and inorganic phosphorus. The most important genes that allow microbes to assimilate and trap phosphorus within their biomass are: *gcd* (quinoprotein glucose dehydrogenase) involved in inorganic phosphate solubilization, *phoD, phoA phoB* (alkaline phosphatase), *appA* (phytase), *phnF, phnE, phnD, phnA* (Carbon-phosphate lyase multi- enzyme complex) implicated in organic phosphate mineralization, *pstS, pstA, pstC, pstB* (phosphate-specific transport systems) for phosphorus transport and uptake, *glpQ* (glycerophosphoryldiester phosphodiesterase) [16,17,18]. Microbes secreting organic acids (e.g., pyruvic acid, gluconic acid) that facilitate the solubilization of phosphate minerals are called phosphate solubilizing bacteria. As these acids are derived from organic carbon compounds via the tricarboxylic acid cycle, there is a close relation between carbon utilization by bacteria and phosphorus availability. Moreover, phosphate dynamics influence the distributions and diversities of phosphate solubilizing microorganisms in the soil [19,20,21,22]. In the soluble inorganic forms, phosphorus exists in soil as phosphodiesters, phosphomonoesters or phytates (in the organic forms) and as metallophosphates [23].

Application of fertilizers, either organic or inorganic, has a direct influence on the microbial communities present in the soil through the nutrients supplied or indirectly via the adjustment of the soil physicochemical properties like the pH. Fertilization affects the activities of soil microbes and nutrient cycling processes and to a greater extent influence the microbial gene expression [24,25]. While an organic fertilizer boosts the abundance, activities, and functions of soil-dwelling microbes, the effects of inorganic fertilizers are expected to be small [26,27,28].

In this context, the aim of the study is to evaluate the effects of soil fertilization with compost and/or inorganic fertilizers on the distribution and abundance of phosphorus cycling genes and rhizosphere microbial community of maize plants.

## 2. Materials and Methods

### 2.1. Collection of Samples and Microbial DNA Extraction

The maize rhizosphere samples were collected from 7-weeks old maize plants as described previously by Enebe and Babalola [29]. The field trial experiment was carried out at North-West University farm and was set up in a complete randomized design (25°47′24.17604″ S, 25°37′9.08328″ E; altitude: 1012 m). The soil type is sandy loam soil. A total of 15 rhizosphere soil samples (5 treatments × 3 replicates per treatment) were collected from the *Zea mays everta* rhizosphere. A total of 15 plots (2 m × 3 m) were used for the study. In a set of three plots each, the soil was treated with 8 ton per hectare compost manure (Cp8), 4 tons per hectare compost manure (Cp4), 60 kg per ha NPK, 120 kg/ha NPK containing urea (N), superphosphate (P_2_O_5_) and muriate of potash (K_2_O) in the ratio of 20:7:3 and another set was unfertilized (Cn0). Both the fertilized and unfertilized plots were planted with maize seeds. The chemical composition of the soil is showed in Table 1, while that of the compost manure are N = 20,045.3 (g/kg), P = (1.0 g/kg), K = 12.3 (g/kg), pH = 7.1. The manure (plant materials and household wastes) was composted for a period of 16 weeks prior to its use. Rhizosphere samples were collected and kept in a sterile plastic bag inside an ice containing box and transported to the laboratory. Plants’ roots and other debris were sieved out using a sieve with 2 mm pore size and the samples were stored at −80 °C for metagenomic shotgun sequencing. The physico-chemical properties of the soil before fertilization and planting as well as the compost manure after the stabilization periods were analyzed according to the basic and standard soil chemical analysis procedure described by Motsara and Roy [30]. This was followed by extraction of the total microbial community DNA from the soil samples (0.25 g) using PowerSoil DNA isolation kit (from MoBio Laboratories, Incorporation, Carlsbad, CA, USA) by following the manufacturer’s guide.

### 2.2. Library Preparation and Sequencing of DNA

The microbial community nucleic acid concentration extracted for the rhizosphere soil samples using PowerSoil kit was examined using Qubit^®^ dsDNA HS Assay Kit (from Life Technologies, Carlsbad, CA, USA) and the deoxyribonucleic acid (DNA) libraries were prepared using Nextera DNA Flex library preparation kit from Illumina Incorporation. The libraries were prepared using 50 nanogram quantity of DNA molecules from each samples according to Nextera library prep protocol. The final concentrations of the libraries prepared were quantified using Qubit^®^ dsDNA HS Assay Kit (from Life Technologies). The libraries average sizes were determined using Agilent 2100 Bioanalyzer analytical machine (from Agilent Technologies, Santa Clara, CA, USA). The DNA libraries, however, were combined in an equal-molar ratios of 0.7 nM. The pooled DNA molecules were sequenced paired end for 300 cycles using the NovaSeq 6000 system machine (from Illumina, San Diego, CA, USA). This DNA sequencing was carried out at the MR DNA laboratory in USA (https://www.mrdnalab.com, accessed on 1 November 2019).

### 2.3. Metagenomic Sequence Analysis

The shotgun sequenced raw reads generated were uploaded into MG-RAST where the reads quality control processes were performed [32]. The pre-processing of the uploaded reads involved the removal of artificial reads, host specific sequences, and other ambiguous base pairs. This was followed by gene annotation using BLAST algorithm [33] and M5NR database [34]. The protein coding-genes annotation were carried out by blasting it in M5NR database and SEED Subsystem level-function and the bacterial families, fungal genus and archaeal genus were generated through blasting the sequences on GenBank (RefSeq). The BlastX was used to perform hit at an e-value cutoff (10^−5^), minimum alignment length (15 base-pairs), and percentage identity (60%). The unidentified sequences were not subjected to further analysis. The MG-RAST normalization tool was applied to enable us cut down on the possible experimental error. The phosphorus cycling genes were curated manually from the total gene files obtained from the SEED Subsystem database at level-function.

### 2.4. Statistical Analysis

The phosphorus cycling genes were evaluated statistically using one-way ANOVA—analysis of variance—at a *p*-value of less than 0.05. The abundance and distribution of bacterial families, fungi, and archaeal genus were visualized using heatmapper (www.heatmapper.ca/expression/, accessed on 1 November 2019). The online software Circos was used in plotting the chart of phosphorus cycling genes, while Simpson, Evenness, and Shannon diversity indices were determined for the samples and contrasted amongst the treatments using Kruskal–Wallis test. Moreover, β diversity was checked using PCoA on the basis of Euclidean distance-matrix. All the analyses were performed with PAST version 3.20 software [35]. Principal Co-ordinate Analysis and principal component analysis (PCA) was performed using CANOCO 5v (Microcomputer Power, Ithaca, NY, USA). The sequences are deposited on NCBI SRA dataset, SRA accession: PRJNA607213.

## 3. Results

### 3.1. Treatments Effect on the Relative Abundance of Bacterial, Fungal and Archaeal Taxa

**Figure 1:**
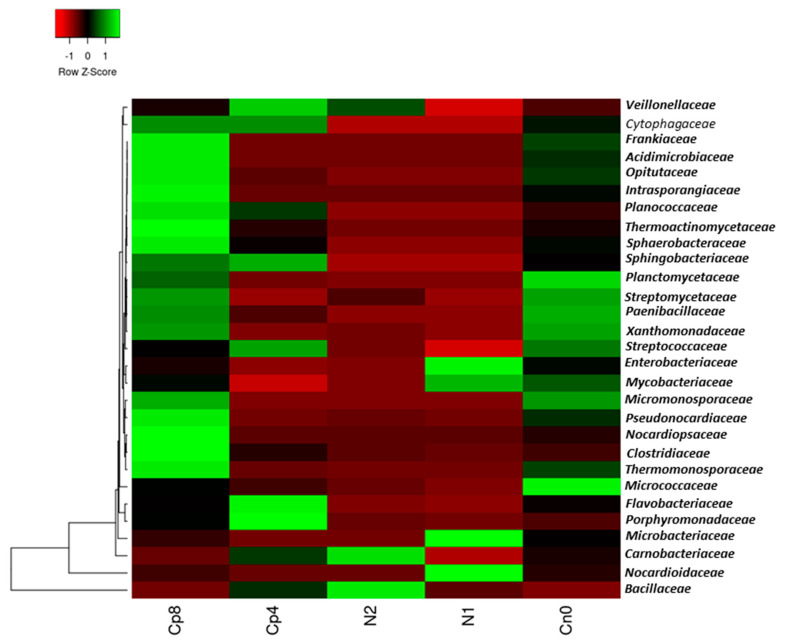
Relative abundance of dominant bacteria families in the maize rhizosphere samples under fertilization and unfertilized soil treatments. Cp8 (8 tons/ha compost manure), Cp4 (4 tons/ha compost manure), N2 (120 kg/ha inorganic fertilizer), N1 (60 kg/ha inorganic fertilizer), and Cn0 (control). The Z-score shows the level of abundance of the bacteria in the various soil samples. The green color represents the most abundant bacterial family, while the red color depicts less abundance within the samples.

The bacterial families present in the samples were Bacillaceae and Carnobacteriaceae which were very abundant in N2, while Pseudonocardiaceae, Clostridiaceae, Cytophagaceae, Micromonosporaceae, Thermomonosporaceae, Nocardiopsaceae, Sphaerobacteraceae, Thermoactinomycetaceae, Planococcaceae, Intrasporangiaceae, Opitutaceae, Acidimicrobiaceae, and Frankiaceae were the most abundant in Cp8. Micrococcaceae and Planctomycetaceae were the most abundant in the untreated control. Nocardioidaceae, Microbacteriaceae, Mycobacteriaceae, and Enterobacteriaceae were the most abundant in N1 and Porphyromonadaceae and Flavobacteriaceae are highly abundant in Cp4 (Figure 1). There was a significant difference in the relative abundance of these bacterial families (*p* < 0.001) within the fertilized and unfertilized maize rhizosphere soil samples.

**Figure 2:**
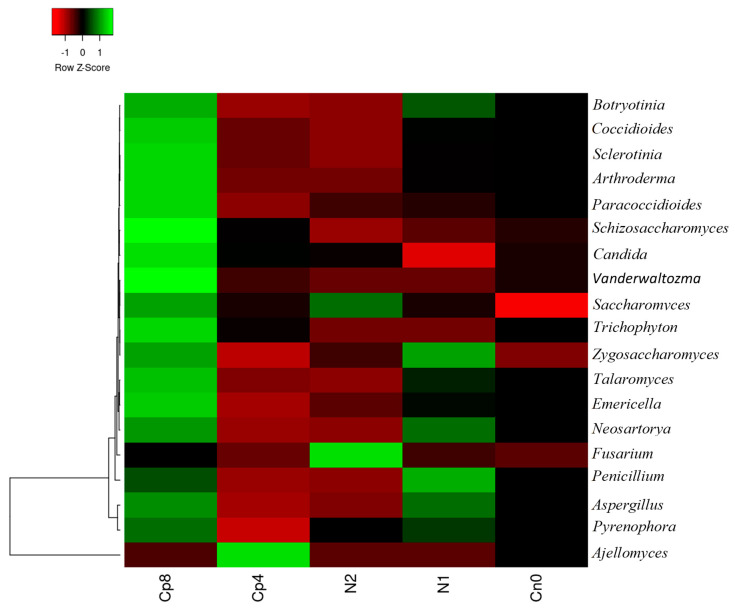
Relative abundance of fungal community at genus level present in the maize rhizosphere under fertilization treatments and the control samples. Cp8 (8 tons/ha compost), Cp4 (4 tons/ha compost manure), N2 (120 kg/ha NPK), N1 (60 kg/ha NPK), and Cn0 (control).

At genus level, with the exception of *Fusarium* and *Ajellomyces*, the rest of the fungi were observed to be more abundant in Cp8. *Botryotinia*, *Aspergillus*, *Pyrenophora*, *Zygosaccharomyces*, *Neosartorya,* and *Penicillium* were also abundant in N1 samples, while *Fusarium* and *Saccharomyces* were dominant in N2. *Ajellomyces* Cp4 (Figure 2). A highly significant difference (*p* < 0.001) was observed within the fertilized treatments and the control. The principal component analysis and principal coordinate analysis of the fungal genus are contained in Figures S1 and S2 and are described in the discussion section.

**Figure 3:**
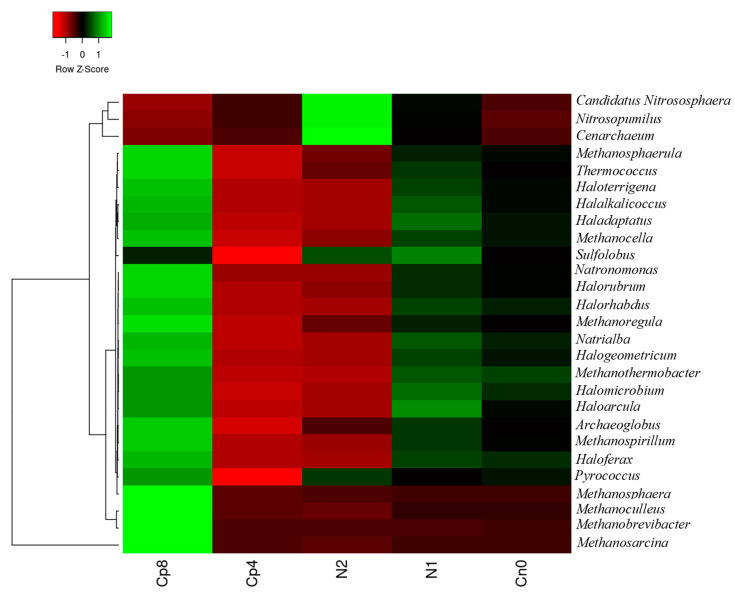
Relative abundance of archaeal community at genus level present in the maize rhizosphere soil under fertilized and unfertilized treatments. Cp8 (8 tons/ha compost), Cp4 (4 tons/ha compost manure), N2 (120 kg/ha NPK), N1 (60 kg/ha NPK), and Cn0 (control).

The archaeal community present in the soil have the highest abundance in Cp8 with the exception of *Cenarchaeum, Candidatus Nitrososphaera,* and *Nitrosopumilus* very abundant in N2 and *Sulfolobus* and *Haloarcula*, which were the most abundant archaea in N1. The control samples (Cn0) have only Methanothermobacter as the most abundant in the rhizosphere soils. The Cp4 treatment have few dominant archaea present in the rhizosphere soil of maize. Finally, there was a significant difference in the abundance levels of archaeal genus (*p* < 0.001) within the inorganic fertilizer, compost manure, and the control soil samples (Figure 3). The principal component analysis and the principal coordinate analysis for the archaeal genus are contained in Figures S3 and S4.

### 3.2. Effects of the Treatments on the Relative Abundance of Phosphorus Cycling Genes

**Figure 4:**
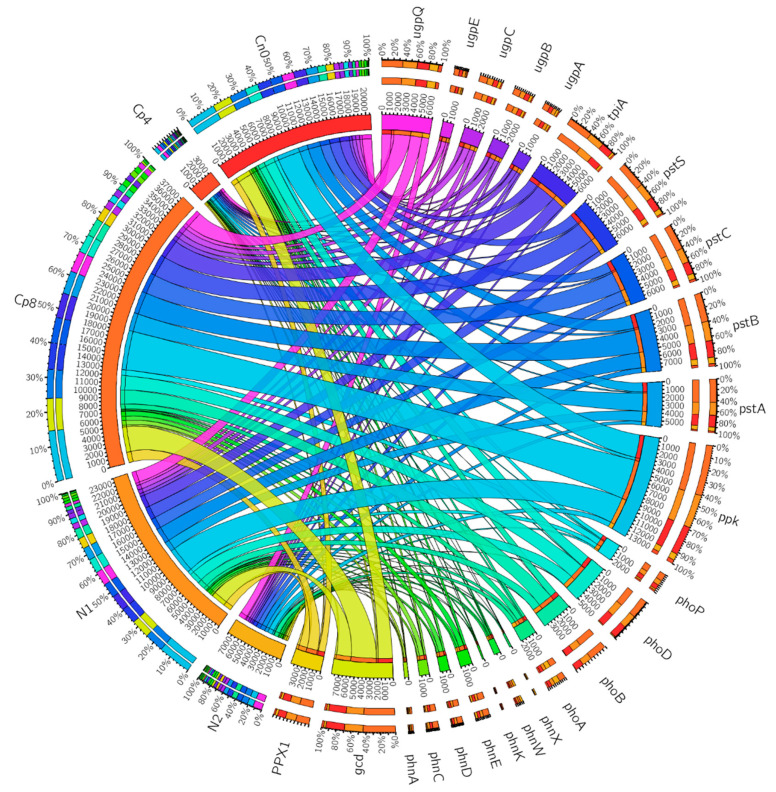
Phosphorous cycling genes relative abundance in the rhizosphere soil samples from maize plants under fertilization and unfertilized conditions as visualized by circus. Cp8 (8 tons/ha compost manure), Cp4 (4 tons/ha compost manure), N2 (120 kg/ha inorganic fertilizer), N1 (60 kg/ha inorganic fertilizer), and Cn0 (control). See Table S1.

Phosphorus cycling genes relative abundance differed significantly (*p* < 0.05) among the fertilization treatments and the control (Table 2). According to Bergkemper et al. [7], phosphorus cycling genes can be categorized as follows: inorganic phosphate solubilizing genes, phytases, phosphoesterase, phosphonate degradation, phosphate transporters, and phosphate starvation regulation genes. The genes that code for enzymes inorganic phosphate solubilization are the most abundant in Cp8, N1, and Cn0, while their relative abundance was least in N2 and Cp4. Polyphosphate kinase (*ppk*), phosphate transporter coding genes (*pstS, pstC, pstB* and *pstA*), triosephosphate isomerase (*tpiA*), quinoprotein glucose dehydrogenase (*gcd*), alkaline phosphatase (*phoD*), and phosphate regulon response regulator (for phosphorus starvation regulation) (*PhoB*) were most abundant in Cp8, N1, and Cn0 treatments. The differences in the abundance of phosphorus cycling genes at the rhizosphere of maize plants under fertilization and unfertilized conditions are highly significant (*p* < 0.05). The gene *ugpQ* (glycerophosphoryl diester phosphodiesterase) was abundant in the treatments Cp8, N1, Cn0, and N2, but least abundant in Cp4, implying that the enzyme possesses a high capability for phosphorus mineralization at the maize rhizosphere under fertilization and unfertilized conditions. The microbial enzyme phosphatases, which initiate catalytic hydrolysis of phosphorus to orthophosphate, a form that plants can assimilate, are richly abundant in the rhizosphere soil samples (Cp8, N1, and Cn0) (Figure 4).

In the circus plot of Figure 4, the phosphorus cycling genes, the control, and the treatments are arranged radially and their relationships shown by the colored chords linking them together. The significance of the relationships between the treatments, control, and the genes are depicted by the size of the chords as seen with *ppk*, *gcd, pstB*, *pstC*, *pstS,* and *tpiA* genes. The chords connecting these genes to Cp8 are bigger in size compared to others. This shows that Cp8 treatment has the most influence on the abundance of phosphorus cycling genes involved in transport, polyphosphate formation, and phosphate metabolism. The size of the arcs, also, reveals the level of the treatments effects on the overall phosphorus cycling genes relative abundance in the soil and it is the summation of all the genes present within each treatment (i.e., the numbers within the inner arcs per treatment). Whereas the length of the arcs for the phosphorus cycling genes are the summation of each genes across the treatments and control. The genes contained in the diagram are: alkaline phosphatase (*phoA*), two-component system, OmpR family, phosphate regulon response regulator PhoB (*phoB*), two-component system, OmpR family, alkaline phosphatase synthesis response regulator PhoP (*phoP*), E3.1.3.1, alkaline phosphatase (*phoB*), glycerophosphoryl diester phosphodiesterase (*ugpQ*), alkaline phosphatase D (*phoD*), phosphate transport system permease protein (*pstC*), phosphate transport system permease protein (*pstA*), phosphate transport system substrate-binding protein (*pstS*), phosphate transport system ATP-binding protein (*pstB*), 4-phytase/acid phosphatase (*appA*), phosphonate transport system permease protein (*phnE*), phosphonate transport system ATP-binding protein (*phnC*), phosphonate transport system substrate-binding protein (*phnD*), phosphonoacetate hydrolase (*phnA*), 2-aminoethylphosphonate-pyruvate transaminase (*phnW*), putative phosphonate transport system ATP-binding protein (*phnK*), putative phosphonate transport system ATP-binding protein (*phnL*), 2-aminoethylphosphonate transport system ATP-binding protein (*phnT*), phosphonoacetaldehyde hydrolase (*phnX*), exopolyphosphatase (*PPX1*), polyphosphate kinase (*ppk*), quinoprotein glucose dehydrogenase (*gcd*), sn-glycerol 3-phosphate transport system permease protein (*ugpA*), sn-glycerol 3-phosphate transport system substrate-binding protein (*ugpB*), sn-glycerol 3-phosphate transport system ATP-binding protein (*ugpC*), sn-glycerol 3-phosphate transport system permease protein (*ugpE*), triosephosphate isomerase (TIM) (*tpiA*).

**Figure 5:**
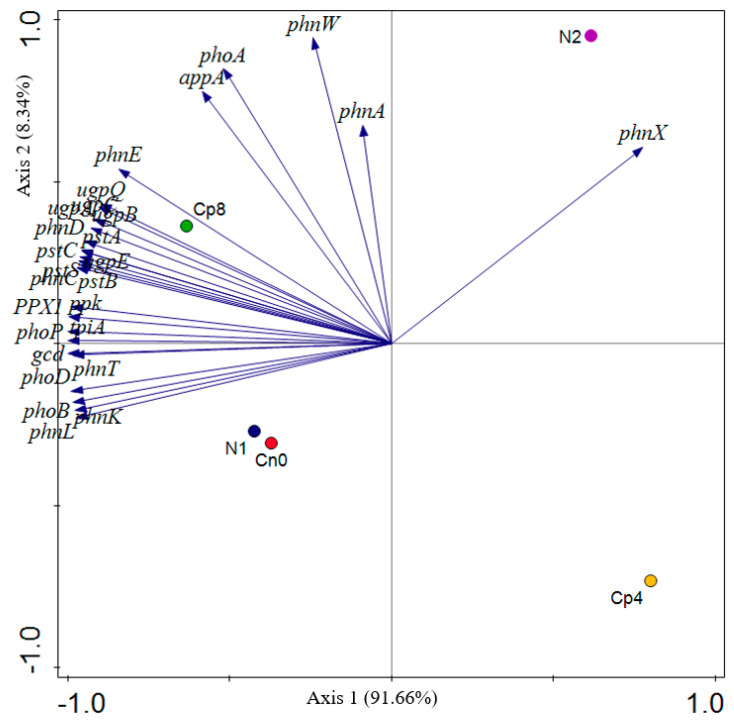
Principal component analysis of the phosphorus cycling genes under fertilization and control treatments within the rhizosphere of maize plants. Cp8 (8 tons/ha compost), Cp4 (4 tons/ha compost manure), N2 (120 kg/ha NPK), N1 (60 kg/ha NPK), and Cn0 (control).

The PCA (principal component analysis) of the phosphorus cycling genes shows that most of the genes were clustered around treatment Cp8, N1, and Cn0 (Figure 5) validating the observation on the abundance and distribution of these genes in Figure 4.

**Figure 6:**
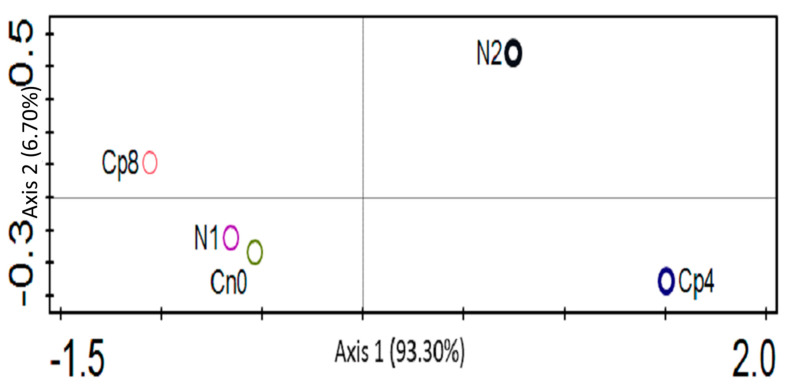
Principal coordinate analysis of the phosphorus cycling genes present at the rhizosphere of maize plants under fertilization and control treatments. Cp8 (8 tons/ha compost), Cp4 (4 tons/ha compost manure), N2 (120 kg/ha NPK), N1 (60 kg/ha NPK), and Cn0 (control).

The α diversity of the phosphorus cycling genes within the treatments are shown by the Shannon, Simpson, and evenness diversity indices. These indices clearly show that there exists a significant difference (Kruskal–Wallis, *p* < 0.001) in the phosphorus genes α diversity (Table 2). The difference in the β diversity was depicted by the principal coordinate analysis, PCoA (Figure 6).

## 4. Discussion

Microbes in the agricultural soil play an active role in mineralization, assimilation, and solubilization of phosphorus containing compounds. Phosphorus is one of essential nutrients for plants and microbial growth. It is a limited nutrient and often occurs in an insoluble form in the soil due to its reactivity with soil minerals [36]. Phosphorus exists in the soil in organic forms as phospholipids, phosphomonoesters, phytates, and phosphodiesters [37,38,39,40,41]. Microbes such as fungi, bacteria, and archaea are involved in the extraction of this nutrient. Due to the scarcity of this nutrient, agriculturists tend to supply it artificially in the form of inorganic and organic fertilization. Soil fertilization has been found to increase bacterial diversity and abundance in the soil [42]. In our metagenomics study, soil fertilization with high quantity of compost manure increased the abundance and diversity of bacteria, fungi, and archaea in the maize rhizosphere compared to other fertilization treatments and control. This is in agreement with the works of Kamaa et al. [43] and Francioli et al. [44] who reported that organic fertilization increased the abundance and diversity of bacteria and fungi in the soil. On the other hand, inorganic fertilization has devastating consequences on the abundance and diversity of microbes present in the soil and on the plant rhizosphere as observed in our study. This observation is supported by the works of Farmer et al. [45] who recorded a decrease in the population and richness of bacteria in the fertilized soil. Moreover, the study by Sapp et al. [46] posited that inorganic fertilizer decreased microbial diversity, while the opposite was observed with organic fertilization. Bacteria belonging to *Frankiaceae, Thermoactinomycetaceae, Streptomycetaceae, Paenibacillaceae,* amongst others, were enriched in the maize rhizosphere fertilized with compost manure (Cp8). These bacterial abundances under the influence of soil fertilization, particularly with high quantity of organic manure, are plant growth promoting microbes that are very beneficial for sustaining plants’ health [47]. They possess genes involved in plant growth promotion, of which phosphorus cycling genes are essential components. A closer look on the organic and inorganic fertilization shows that the abundance and distributions of bacteria, fungi, and archaea differed in response to the types and quantity of fertilizers used, as presented in Figure 1, Figure 2 and Figure 3. To further reveal the fertilizer’s types and quantity influence on the microbial community, the principal component analysis explanation will suffice. At the negative axis 1 of the principal component analysis (Figure S1), there is a strong negative loading for the fungi *Coccidioides*, *Talaromyce*, *Aspergillus*, *Sclerotinia*, *Penicillum* etc., and a weak positive loading for *Fusarium* fungi at positive axis 1. This implies that soil fertilization with compost and/or low inorganic fertilizer could trigger the proliferation of diverse communities of fungi that respond to the added nutrients [48] in the soil at negative axis 1. Organic fertilizers are known to increase the diversity of soil microbial communities [49,50,51] as it supplies the required nutrients for microbial growth and metabolism, thereby increasing soil microbial richness [45].

In axis 2, there is a strong positive loading for *Fusarium* fungi. This reflects the impact of inorganic fertilization at a high application dose on the reduction in fungi diversity and promotion of *Fusarium* capable of withstanding fertilizer-induced soil acidification [52]. *Fusarium* fungi is a known plant disease-causing agent and our result has shown that nitrogen fertilization at a high dose could result in the development of plant diseases caused by *Fusarium* pathogens [53]. *Ajellomyces* and *Schizosaccharomyces* have strong negative loading at axis 2. This reflects that soil under organic fertilization could promote the proliferation of non-plant disease-causing fungi that participate in nutrient biogeochemistry [54,55]. Figure S2 shows the β diversity of fungi across the treatments and control samples. Therefore, soil fertilization supports an abundance of fungi more than the control. Fertilization of soil with organic and/or inorganic fertilizers has shown to influence the abundance and distribution of phosphorus cycling genes within the maize rhizosphere. The obtained metagenomes from the compost treated (Cp8 and Cp4), inorganic fertilized (N2 and N1), and control (Cn0) samples have genes encoding for phosphoesterase, inorganic phosphate solubilization, phosphate transport, degradation of phosphonate, and starvation sensitive phosphate regulation genes. Our study showed that the genes involved in phosphorus cycling at the maize rhizosphere were in high abundance. Genes implicated in inorganic phosphate solubilization were also high and this indicates that the microbes present at the rhizosphere have the capacity to utilize inorganic phosphorus. Alkaline phosphatase (*phoD*) was positively increased by the compost (high dose), which is in agreement with Fraser et al. [56] as well as in inorganic fertilizer (low dose) and the control.

However, the principle of nitrogen–phosphorus ratio stoichiometry, which states that increase in nitrogen addition enhances microbial inorganic phosphorus demand [57], was observed in our study to an extent, especially at the low inorganic fertilizer dose (N1 −60 kg N/ha), which increased the abundance of the phosphorus cycling genes, but did not hold true at the very high dose of 120 kg N/ha (N2) treatment. The only gene increased was *ugpQ* (glycerolphosphoryl diester phosphodiesterase), coding an alkaline phosphatase capable of catalyzing the breakdown of phospholipid (glycerolphosphodiesters) to generate glycerol 3 phosphate and alcohol [58]. High dose of inorganic fertilizer does have repressive effects on the abundance of phosphate starvation regulation genes, phosphodiesterase, phosphonate degradation, inorganic phosphate solubilization, and phosphorus transport genes (Figure 4). This implies that assimilation, solubilization, transformation, and transport of phosphorus were impaired by high doses of inorganic nitrogen fertilizer, despite a slight enhancement of the abundance of *ugpQ* genes. This observation could be as a result of inorganic fertilizer associated acidification effects that suppress the viability and activities of the rhizosphere bacterial community [59]. To enhance organic phosphorus mineralization by microbes, inorganic nitrogen fertilizer application should be at a quantity suitable to enhance the microbial extraction of phosphorus from the organic compounds [60]. Surprisingly, low doses of compost manure have the same repressive effects on the phosphorus cycling genes as do the high doses of inorganic nitrogen fertilizer, therefore, further investigation is needed to understand the rationale behind this observation.

The *phnX, phnW*, *phoA*, *appA,* and *phnA* genes (in Figure 5) are the major drivers to separate the N2 from the control and treated samples. The factors along the negative values of axis 1 distinguished the Cp8 from the N1 and Cn0 samples. Axis 1of the principal component analysis of the phosphorus cycling genes shows a strong positive loading for *phnX* (phosphonoacetaldehyde hydrolase) and strong negative loadings for *pstC, pstB, ppk, phoD, gcd, pstA* etc. The analysis of these genes reflects strong organic phosphate mineralization through the breakdown of carbon–phosphorus (C-P) bonds to yield phosphate ions and acetaldehyde by the enzyme phosphonoacetaldehyde hydrolase [61] in response to soil fertilization at positive axis 1 and increasing phosphate transport, polyphosphate formation, and mineralization of soil organic phosphorus at negative axis 1 [7,62,63]. At axis 2, there is a strong positive loading for alkaline phosphatase (*phoA*), 2-aminoethylphosphonate-pyruvate transaminase (*phnW*), and phosphonoacetate hydrolase (*phnA*). Alkaline phosphatase, a periplasmic enzyme with magnesium and zinc ions as cofactor is a thermostable and protease resistant enzyme [64] responsible for the uptake of inorganic phosphorus in a phosphorus limited environment [65,66]. The gene product of *phnW* is involved in bacterial mineralization of 2-aminoethylphosphonic acid through phosphonatase pathway. The substrate serves as a carbon, nitrogen, and phosphorus source for the microbes [67,68], whereas the *phnA* gene product (phosphonoacetate hydrolase) catalyzes the conversion of phosphonoacetate to acetate and phosphate ions through the cleavage of carbon–phosphorus bonds. The acetate produced serves as the sole carbon source for the microbes in the soil [69,70]. They are key enzymes that participate in phosphorus mineralization, solubilization, and uptake by microbes in the maize rhizosphere.

Our study also demonstrated that the most abundant phosphorus cycling genes present at the maize rhizosphere under compost (Cp8), inorganic fertilizer (N1), and control (Cn0) treatments is *ppk* (polyphosphate kinase), which catalyzes the polymerization of phosphorus monomers to generate polyphosphate molecules. Polyphosphate molecules serve as energy reservoirs in microbes for biochemical processes involving phosphorylation of biomolecules like sugars, nucleic acid, proteins etc., and enhance their survival and growth in the environment [71,72]. The formation of biofilm, sequestration of cations, expression of genes and signaling are among the biological roles of polyphosphate molecules in a microbial cell [73]. The second most abundant gene family is *pstSBAC* (the high-affinity-phosphate transporters), which facilitate the assimilation of phosphorus from the soil. There is a relationship between the polyphosphate kinase genes abundance and the transporters. For polyphosphate to be formed, phosphate transporters must enhance the acquisition of these phosphate molecules from the environment. Therefore, high abundance of *ppk* genes and *pstSBAC* reflected that there was high microbial capacity for the assimilation of phosphorus in the treated maize rhizosphere. At high inorganic fertilization, the genes implicated in transport, uptake, and solubilization of phosphorus were decreased, which is in agreement with the works of Bergkemper et al. [7] and Ikoyi et al. [74].

Moreover, *gcd* (quinoprotein glucose dehydrogenase) was increased in abundance by the high compost, low inorganic nitrogen fertilizer, and the control treatments. The enzyme synthesized by this gene is paramount in inorganic phosphate metabolism due to its catalytic conversion of glucose molecules to gluconic acid using a prosthetic group cofactor, pyrroloquinoline quinone [63,75], thereby regulating as well as enhancing the solubilization of trapped inorganic phosphorus in the soil.

## 5. Conclusions

In summary, soil fertilization with both organic manure (compost derived from domestic waste and plant materials) and low quantities of inorganic nitrogen fertilizer have nearly the same effects as maize plants in promoting the abundance of genes involved in the phosphorus cycle. Genes such as *ppk* involved in polyphosphate formation and *pstSABC* (for phosphate transportation across the cell membrane) are highly enriched in these treatments. These genes facilitate phosphorus immobilization. At high doses of inorganic fertilizer application or low compost manure treatments, the phosphorus cycling genes were repressed and their abundance decreased. Evidence presented so far has shown that organic fertilizers are the best source of nutrients for the promotion of soil microbial abundance, diversity, and functions. Our study has also brought to light the basis for the manipulation of the rhizosphere microbial community and their genes to improve availability of phosphorus and in phosphorus cycle regulation in agro-ecosystems.

## Figures and Tables

**Table 1:** Physicochemical properties of the soil prior to planting, and fertilization.

Soil Property	Value
Physical characteristics	
% Sand	80
% Silt	5
% Clay	15
Chemical properties	
pH (1:2.5 water)	4.97
Total Nitrogen (mg/kg)	377
Total Bray 1 Phosphorus (mg/kg)	100.5
Total potassium (mg/kg)	285
Total calcium (mg/kg)	388
Total magnesium (mg/kg)	162
Total sodium (mg/kg)	5
% carbon	0.36
S-Value (sum of extractable Ca, Mg, K and Na) (cmol(+)/kg)	4.59
% calcium	48.0
% magnesium	33.2
% potassium	18.3
% sodium	0.5
Extractable acidity (me %)	0.03

Adapted from Enebe and Babalola [31].

**Table 2:** Diversity indices of the phosphorus cycling genes within the rhizosphere of maize plants under treatments.

Diversity Indices.	Cp8	Cp4	N2	N1	Cn0
Simpson_1-D	0.9278	0.9375	0.9338	0.9259	0.9266
Shannon_H	2.82	2.933	2.885	2.798	2.811
Evenness_eH/S	0.699	0.7827	0.7458	0.6836	0.693

## Data Availability

The data is deposited at NCBI SRA under the accession number: PRJNA607213.

## Supplementary Materials

The following are available online at https://www.mdpi.com/article/10.3390/genes12071022/s1, Figure S1: PCA for the fungal genus dominant at the maize rhizosphere under fertilization and control. Figure S2: PCoA analysis for the fungal genus dominant at the maize rhizosphere under control and soil fertilization. Figure S3: Principal component analysis of archaeal genus present at the maize rhizosphere under control, organic and inorganic fertilization. Figure S4: Principal coordinate analysis of the archaeal genus abundant in the maize rhizosphere under fertilization and control conditions. Table S1: Quantity of microbial phosphorus cycling genes present in the rhizosphere soil samples.
