# Supplementary material for: The Influence of Soil Fertilization on the Distribution and Diversity of Phosphorus Cycling Genes and Microbes Community of Maize Rhizosphere Using Shotgun Metagenomics
Source: Genes (Basel). 2021 Jun 30;12(7):1022. doi: 10.3390/genes12071022 (PMC8306440; doi:10.3390/genes12071022)
Supplement: Supplementary file 1 [file genes-12-01022-s001.zip › genes-1213064-supplementary.pdf]

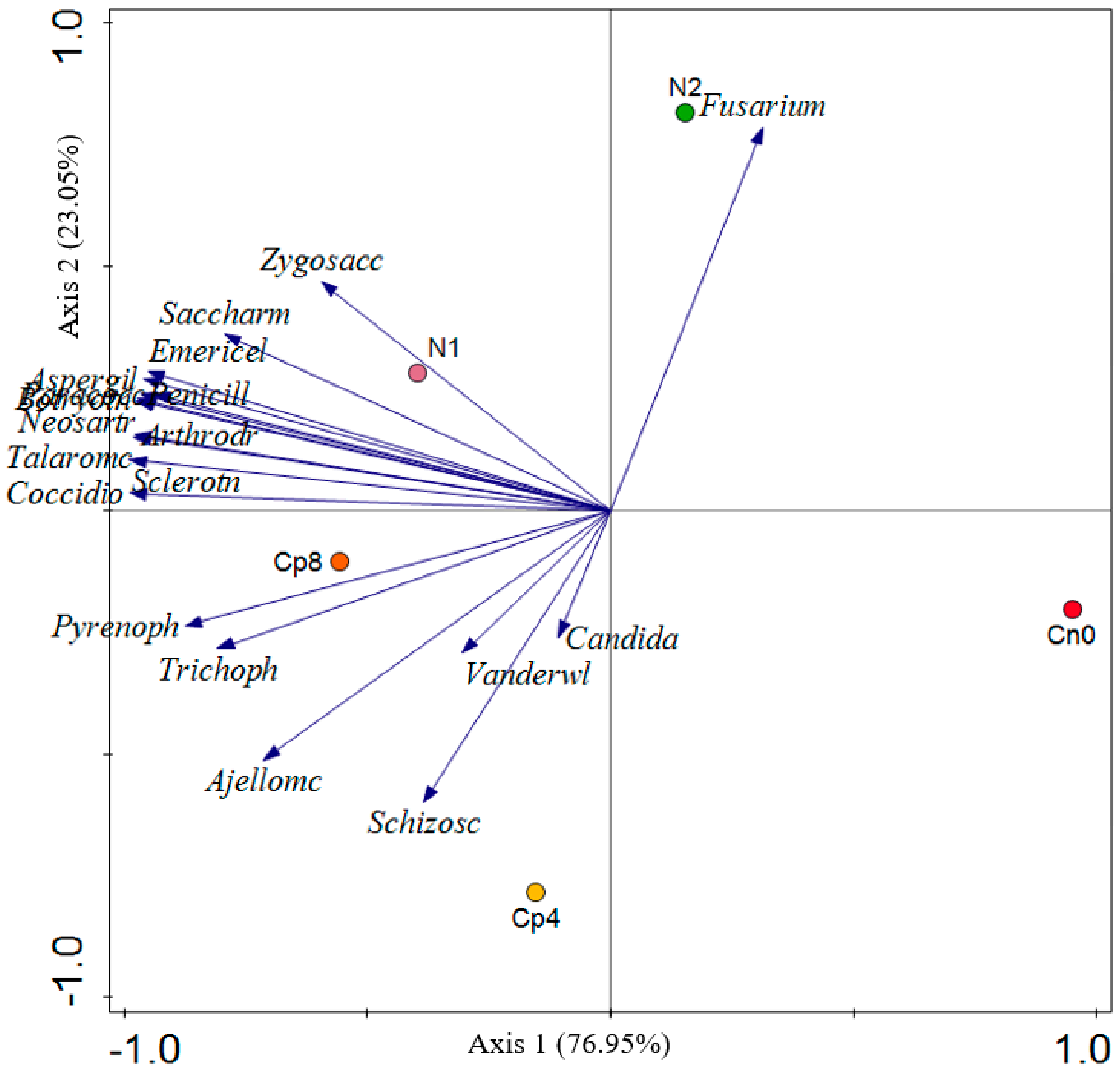

Figure S1. PCA for the fungal genus dominant at the maize rhizosphere under fertilization and control.

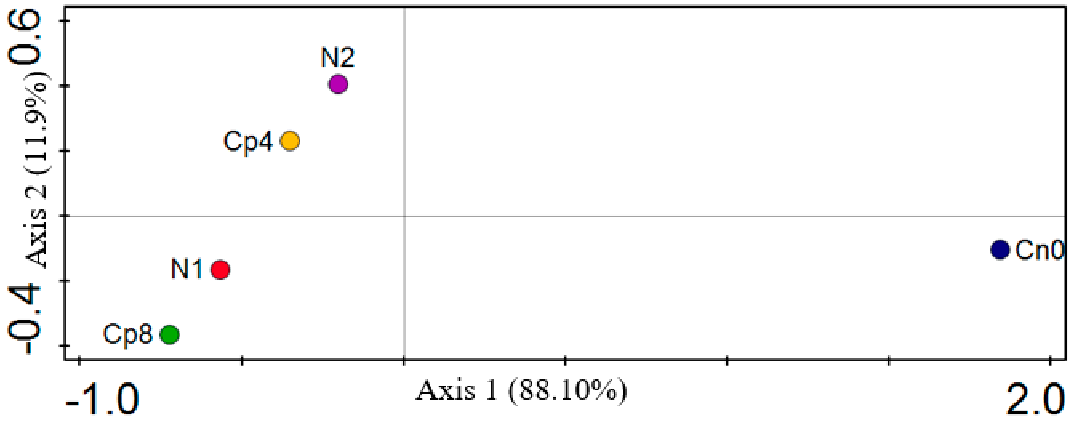

Figure S2. PCoA analysis for the fungal genus dominant at the maize rhizosphere under control and soil fertilization.

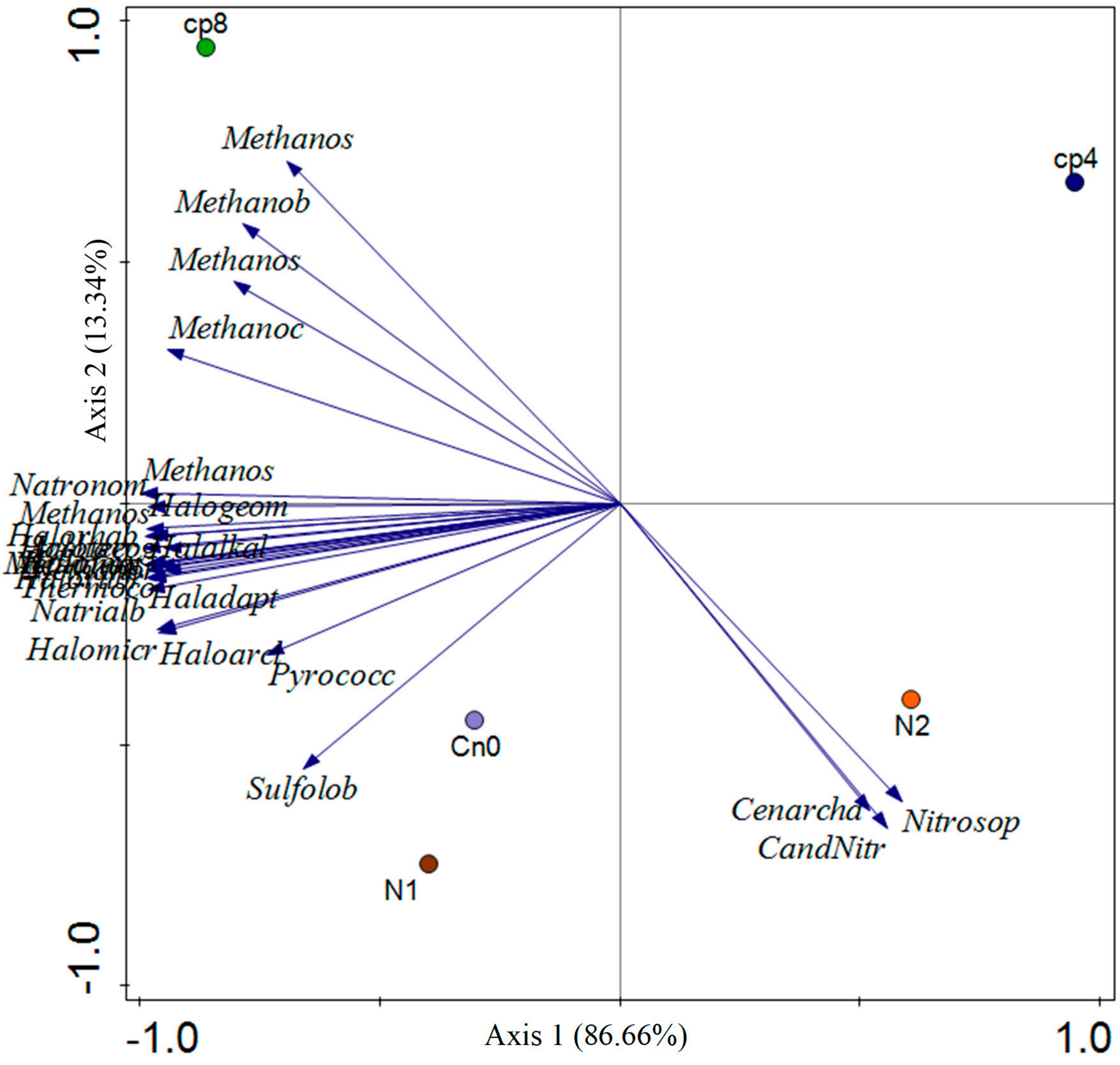

Figure S3. Principal component analysis of archaeal genus present at the maize rhizosphere under control, organic and inorganic fertilization.

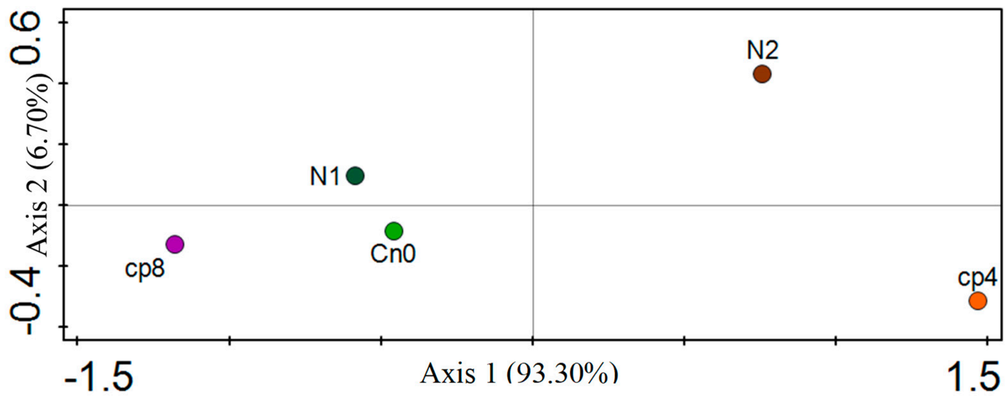

Figure S4. Principal coordinate analysis of the archaeal genus abundant in the maize rhizosphere under fertilization and control conditions.

Table S1. Quantity of microbial phosphorus cycling genes present in the rhizosphere soil samples.

function	Cp8	Cp4	N2	N1	Cn0
phoA	704	173	575	389	347
phoB	1941	29	13	1078	920
phoP	779	144	183	565	513
ugpQ	2285	367	968	1578	1324
phoD	2002	79	69	1512	1455
pstC	2759	230	597	1596	1332
pstA	2501	151	470	1392	1259
pstS	2854	186	547	1777	1425
pstB	3132	268	676	2108	1801
appA	3	1	3	2	2
phnE	679	54	238	314	300
phnC	620	49	104	270	278
phnD	597	39	111	278	253
phnA	231	76	130	55	59
phnW	200	103	241	170	138
phnK	93	2	1	63	58
phnL	79	1	1	62	54
phnT	32	1	1	34	37
phnX	22	40	128	16	23
PPX1	1472	155	279	1134	890
ppk	5471	391	696	3639	3153
gcd	3492	203	249	2016	2004
ugpA	639	109	273	405	414
ugpB	761	114	297	552	515
ugpC	875	132	349	646	532
ugpE	561	64	144	342	344
tpiA	2683	174	234	1686	1318
